# Maternal health study: a prospective cohort study of nulliparous women recruited in early pregnancy

**DOI:** 10.1186/1471-2393-6-12

**Published:** 2006-04-11

**Authors:** Stephanie J Brown, Judith M Lumley, Ellie A McDonald, Ann H Krastev, collaboration with the Maternal Health Study collaborative group

**Affiliations:** 1 Mother and Child Health Research, La Trobe University, 251 Faraday Street, Carlton, Victoria 3053, Australia

## Abstract

**Background:**

In the first year after childbirth, 94% of women experience one or more major health problems (urinary incontinence, faecal incontinence, perineal pain, back pain). Difficulties in intimate partner relationships and changes affecting sexual health are also common. The aim of this study is to investigate changes in women's health from early pregnancy until four years after the birth of a first child.

**Methods/design:**

The Maternal Health Study is a longitudinal study designed to fill in some of the gaps in current research evidence regarding women's physical and psychological health and recovery after childbirth. A prospective pregnancy cohort of >1500 nulliparous women has been recruited in early pregnancy at six metropolitan public hospitals in Melbourne, Australia between April 2003 and December 2005. In the first phase of the study participants are being followed up at 30–32 weeks gestation in pregnancy, and at three, six, nine, 12 and 18 months postpartum using a combination of self-administered questionnaires and telephone interviews. Women consenting to extended follow-up (phase 2) will be followed up six and 12 months after any subsequent births and when their first child is four years old. Study instruments incorporate assessment of the frequency and severity of urinary and bowel symptoms, sexual health issues, perineal and abdominal pain, depression and intimate partner violence. Pregnancy and birth outcome data will be obtained by review of hospital case notes.

**Discussion:**

Features of the study which distinguish it from prior research include: the capacity to identify incident cases of morbidity and clustering of health problems; a large enough sample to detect clinically important differences in maternal health outcomes associated with the method of birth; careful exposure measurement involving manual abstraction of data from medical records in order to explore mediating factors and possible causal pathways; and use of a variety of strategies to improve ascertainment of health outcomes.

## Background

In the 1990s two large hospital cohort studies in the UK and a population-based Australian cohort study drew attention to the significant burden of physical ill health among women in the year after giving birth [1-3]. Common experiences highlighted by these studies include back pain, exhaustion, urinary and anal incontinence, constipation, haemorrhoids and persisting perineal pain. There is also mounting evidence regarding changes affecting women's sexual health during and after pregnancy [3-5], and about the impact of intimate partner violence on childbearing women [6-8].

This research heightened awareness of the extent of maternal morbidity after childbirth and contributed to debate concerning the contribution of pregnancy and birth events to longer term health outcomes, such as prolonged perineal pain and dyspareunia, urethral and anal sphincter damage, genital prolapse, and urinary and anal incontinence [9-15]. There is now a growing literature comparing maternal health outcomes following spontaneous and operative vaginal births with elective and emergency caesarean section.

Despite an increasing number of studies examining obstetric risk factors for individual morbidities such as urinary and anal incontinence, evidence concerning risks associated with operative vaginal birth and caesarean section remains inconclusive. Limitations of the existing literature include: lack of study power for assessing key obstetric exposures (e.g. method of birth) or mediating factors (e.g. perineal trauma); study designs that do not facilitate reliable identification of incident cases, i.e. the onset of symptoms during pregnancy or the first few months after giving birth; inadequate follow-up periods (few studies extend beyond 6–12 months postpartum); and a tendency to focus on individual morbidities, such as urinary incontinence or anal incontinence, ruling out assessment of combined morbidities or a more comprehensive assessment of women's general health following childbirth.

The Maternal Health Study is a longitudinal cohort study investigating the health and well-being of nulliparous women during pregnancy and after the birth of their first child. Recruitment to the study commenced in 2003 and was completed in December 2005. The study is designed to address gaps in current research evidence regarding women's physical and psychological health and recovery after childbirth. In the first phase of the study women are being followed from early pregnancy (≤20 weeks gestation) through to 18 months postpartum. Women consenting to participate in extended follow-up (phase 2) will be followed up six and twelve months after any subsequent births, and when their first child is four years old. This paper outlines the study methods and design considerations for phase 1 of the study.

## Methods/design

### Aims

Specific aims in phase 1 of the study are:

1. To measure the incidence and natural history (onset, severity, and duration) of maternal health problems, in particular urinary incontinence, anal incontinence, perineal pain, sexual health issues, and depression among women having their first child.

2. To explore the contribution of obstetric risk factors; in particular obstructed labour, method of birth, and degree of perineal trauma, to postpartum health problems, taking into account early pregnancy health status.

3. To investigate reasons for non-disclosure of maternal health problems, and for the limited use of primary and specialist health services for specific postpartum health issues.

### Study hypotheses

Pre-specified study hypotheses are:

1. That vaginal birth assisted by forceps or vacuum extraction is an independent risk factor for incident cases of urinary incontinence, anal incontinence, persisting perineal pain and sexual problems after birth.

2. That both mediolateral episiotomy and midline episiotomy are independent risk factors for incident cases of urinary incontinence, anal incontinence, persisting perineal pain and sexual problems after spontaneous vaginal birth.

3. That mediating factors for urinary incontinence, anal incontinence, persisting perineal pain and sexual problems after spontaneous and assisted vaginal births include fetal size (≥ 4000 grams), maternal size (≤ 150 cm, BMI ≥ 30), length of labour, partogram evidence of obstructed labour, lithotomy position or upright position of the mother during late labour and birth, and non-suturing of perineal tears.

4. That these mediating factors will differ in their strength of association with urinary incontinence, anal incontinence, persisting perineal pain and sexual problems leading to new hypotheses about causal pathways.

5. That the number and severity of physical health problems in the year after birth will increase the probability of women becoming depressed or remaining depressed.

6. That socio-economic factors such as low income, single marital status and non-English speaking background will increase the probability of women becoming depressed or remaining depressed, taking obstetric history into account.

7. That women who have not disclosed health problems to their GP or specialist obstetrician will be willing to discuss the reasons for non-disclosure in a telephone interview.

8. That the reasons for non-disclosure will include: the perception that the problem is 'natural', to be expected after childbirth, too embarrassing to discuss, not a medical problem, or that nothing can be done about it; fear of medical tests and investigations; fear of surgery; fear that treatment might make it worse; fear that treatment might create new problems; fear that the problem will not be regarded as a significant health issue by health professionals.

9. That socio-economic factors such as low income, single marital status, and non-English speaking background will decrease the probability of women experiencing physical and emotional health problems disclosing these health problems to primary and specialist health professionals.

### Sample

Recruitment of nulliparous women booking to give birth at five metropolitan public hospitals in Melbourne, Australia commenced in April 2003. Participating hospitals have a mix of high and low risk perinatal services. Two hospitals are tertiary level centres with neonatal intensive care (NICU) facilities.

Eligibility criteria for the cohort are: age ≥18 years; nulliparity; estimated gestation 10–20 weeks of pregnancy (according to date of last menstrual period or ultrasound) at enrolment; sufficient proficiency in English to complete telephone interviews and written questionnaires. Criteria for exclusion after study enrolment are: spontaneous abortion prior to 20 weeks gestation; ectopic pregnancy; termination of pregnancy prior to 20 weeks gestation. Women who experience a multiple pregnancy, a stillbirth or neonatal death or a serious maternal illness (e.g. psychotic illness, severe pre-eclampsia) will not be excluded from participation in the cohort, but may be excluded from some analyses.

### Sample size

Sample size calculations were conducted using Epi Info Version 5 based on prevalence estimates for primary outcomes derived from a population-based Victorian survey of recent mothers [16]. Table 1 shows power calculations for the study with alpha of 0.5 and beta of 0.20, assuming ratios of:

**Table 1:** Sample size calculations

**Exposure comparison (ratio of unexposed: exposed)**	**Outcome of interest**	**Difference**	**Sample size estimate**	Total number required*
**Spontaneous vaginal birth:forceps/vacuum extraction (2:1)**	Urinary incontinence Faecal incontinence Sexual problems Perineal pain	11% versus 18% 2.6% versus 8% 24% versus39% 20% versus 53%	930 642 360 168	1200 800 450 210
**Emergency caesarean:forceps/vacuum extraction (1:2)**	Urinary incontinence Faecal incontinence Sexual problems	7% versus 18% 3.9% versus 10.5% 29% versus 39%	371 573 841	950 1500 2200
**Elective caesarean:forceps/vacuum extraction (1:5)**	Urinary incontinence Faecal incontinence Sexual problems	2.4% versus 18% 2.8% versus 10.5% 27% versus 39%	276 763 938	900 2500 3100

* Taking into account the relative frequency of different methods of delivery

• 2:1 (unexposed:exposed) for comparison of spontaneous vaginal births with operative vaginal births;

• 1:2 for comparison of emergency caesarean sections with operative vaginal births; and

• 1:5 for comparison of elective caesarean sections with operative vaginal births.

These ratios were based on routinely collected data for women giving birth in Victoria in 1999. Since the five study hospitals are all public hospitals and nulliparity was an eligibility criterion for the study, the ratios were calculated based on confinements of women admitted as public patients for the birth of their first child.

Using these parameters and allowing for 20% loss to follow-up by 18 months postpartum, we estimated that a sample of 1,900 women would be sufficient to test the hypothesis that primiparous women having an operative vaginal birth have a twofold greater relative risk of developing urinary incontinence, faecal incontinence and sexual problems compared with women having a spontaneous vaginal birth, and to detect a relative risk of 2.0 for urinary incontinence for all exposure comparisons.

#### Recruitment procedures

The estimated number of women giving birth at the five study hospitals prior to study commencement was 12,700 per annum (based on confinements in 1998/99). At the two tertiary level hospitals (accounting for 8,000 births per annum) approximately 40% of births are to women born overseas of non English speaking background, about half of whom were expected to have insufficient proficiency in English to fulfil the eligibility criteria. A minority of confinements at participating hospitals in 1998/99 (2.1%) were to women receiving care as private patients. Based on these figures and allowing for 10% of women booking after 18 weeks gestation (overlapping to a degree with non-English speaking women), we anticipated it would take approximately 20 months to recruit the cohort assuming a response fraction of around 40%.

The main method of recruitment is via mailed invitation facilitated by the study hospitals. Procedures have been established at each hospital to facilitate: (i) identification of eligible women using hospital data systems; (ii) regular weekly or fortnightly mail-outs to eligible women booking in the preceding week or fortnight; and (iii) weekly or fortnightly follow-up reminder cards for women who have not enrolled two weeks after the original mail-out. The invitation package contains: (i) a brief information leaflet explaining the purpose of the study and inviting women to take part; (ii) a plain language statement with further detail about the study and study consent forms; (iii) the first study questionnaire (Q1); (iv) a sheet for recording contact details, and (v) a reply paid envelope for returning the questionnaire, study consent form and contact details to the research team based at Mother & Child Health Research (MCHR), La Trobe University. The information package includes the names and phone numbers of research team members for queries, and gives the contact number for hospital and/or the La Trobe University Ethics Committee, in case any recipient wished to obtain more information or make a complaint. Recruitment procedures were piloted at each study hospital in late 2002 and early 2003. Different procedures for identifying eligible women and handling the mailing out of the invitation packages and reminders were developed to suit each site.

### Follow-up procedures

Following receipt of the baseline questionnaire (Q1) and signed consent form women are mailed a study postcard which acknowledges receipt of the first questionnaire, thanks them for joining the study, and provides a further copy of contact phone numbers for members of the research team. Participating hospitals are notified of all new enrolments, in order for this information to be flagged in hospital data systems. Information regarding births to study participants or any relevant adverse events (e.g. pregnancy losses, admission of baby to NICU, neonatal deaths) is provided by each study hospital to the project data manager on a regular basis.

**Figure 1:**
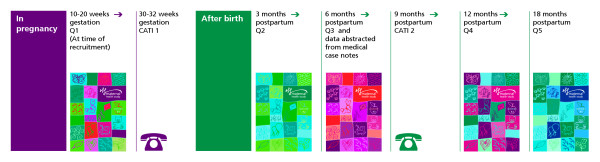
Flow chart summarising data collection in phase 1 of the Maternal Health Study.

All contact with study participants after enrolment is managed by the research team based at MCHR. In phase 1 of the study, follow-up is scheduled to occur at: 30–32 weeks gestation, and at three, six, nine, 12 and 18 months postpartum using a combination of self-administered questionnaires and telephone interviews (see Figure 1). Participant contact details and the names, addresses and phone numbers of alternate contact/s are checked and updated at each telephone interview (30–32 weeks gestation, and at nine months postpartum).

At each questionnaire follow-up, study participants are mailed a copy of the relevant questionnaire, a covering letter, and a reply paid envelope for returning the questionnaire to the research team. Mailed reminders are sent to women who have not returned a scheduled follow-up questionnaire two weeks after mail-outs at three, six, 12 and 18 months postpartum. Further follow-up of women who have not returned a questionnaire four weeks after the original mailing is undertaken by phone by one of the two project co-ordinators.

Telephone interviews at 30–32 weeks gestation and nine months postpartum are conducted by a small team of trained female interviewers using computer assisted telephone interview techniques (CATI). Interviews are arranged at times to suit participants, with up to eight attempts made to contact women in order to complete an interview.

### Ethical issues

The key ethical issues in this study are:

• a worthwhile question in terms of the size of the population affected, and associated morbidity, quality of life and resource implications

• informed consent to participation in the study, and for information to be obtained from hospital medical records, with the proviso that consent may be withdrawn at any time, and that care provided by hospitals and other agencies will not be influenced by decisions about participation

• an adequate sample size to detect clinically important differences in outcome associated with the method of birth

• acceptability of the study instruments and study procedures to participants

• safe-guarding confidentiality of data.

Study procedures for inviting women to participate were designed to give women detailed information about all aspects of the study. As some of the issues covered in study questions are potentially of a personal or sensitive nature, we wanted to ensure that women were made aware of this before consenting to take part. The first study questionnaire which covers symptoms during early pregnancy and prior to pregnancy was included in the invitation package so that women would be fully acquainted with the types of issues covered by the study before giving their written consent to participate. Study materials emphasised that participants were welcome not to answer particular questions if that was their preference, and the telephone interviewers were trained to reinforce this information prior to commencing each interview.

Study procedures, information materials, self-administered questionnaires and interview schedules were piloted to assess acceptability and relevance, and to ensure that any problems relating to the clarity of instructions and explanations were sorted out prior to commencing each stage of follow-up.

Signed consent was sought separately for information to be obtained from individual hospital medical records regarding pregnancy complications, events during labour and birth, and any postnatal complications, including re-attendances at the study hospital after discharge.

A detailed study protocol was developed outlining procedures for managing recruitment and follow-up (available from the investigators on request), which also became the manual for training research staff working on the study. A separate written study protocol was developed as a guide for research staff conducting telephone interviews. All interviewers are female, and women were informed of this in information materials prior to joining the study.

Procedures for safeguarding the confidentiality of participants include: storage of questionnaires and medical records data abstraction forms in a secure location; separation of names and contact details from data, password protected computer access. All staff working on the study are required to sign a statement confirming that they will adhere to study procedures regarding confidentiality.

Ethics approval for the study has been granted by the La Trobe University Human Research Ethics Committee, and by the Human Research Ethics Committees of the Royal Women's Hospital, Southern Health and Angliss Hospital.

### Data collection

A combination of mailed questionnaires and telephone interviews is being used to maximise disclosure of health problems during pregnancy and follow-up. Study instruments incorporate assessment of the frequency and severity of urinary and bowel symptoms, sexual health issues, perineal and abdominal pain, depression and intimate partner violence using previously validated standardised questions where possible. Questions about urinary symptoms (stress, urge and mixed incontinence) are based on instruments previously validated in Australian, Scandinavian and UK populations [17-20]. Assessment of bowel symptoms (leakage of solid or liquid stool, faecal urgency) is based on questions adapted from validated instruments used in Australian community prevalence studies [21-23]. Items on sexual health include questions previously used in the Australian Study of Health and Relationships [24], the Longitudinal Australian Women's Health Study [25] and a recent UK study of women's sexual health after childbirth [5]. Postnatal pain is being assessed using questions adapted from the McGill Pain Questionnaire [26], and the Brief Pain Inventory [27].

The SF-36 is incorporated in questionnaires in early pregnancy, and at six and 12 months postpartum to provide a general measure of health and well-being to be compared with reporting of specific health problems [28]. Assessment of emotional well-being is being made using the Edinburgh Postnatal Depression Scale (EPDS) and the mental health domain of the SF-36. The EPDS is incorporated in questionnaires in early pregnancy and at three, six, 12, and 18 months postpartum. The EPDS was developed to avoid problems with somatic and social items (eg. change of appetite, sleep disturbance) in other standard measures of depression that cannot be interpreted as morbidity in women with a small baby. It has been found to have good sensitivity and specificity when assessed against psychiatric diagnosis of depression in three studies conducted in the UK, and a small Australian validation study [29,30].

The questionnaire at 12 months postpartum incorporates a validated scale to assess intimate partner abuse (shortened Composite Abuse Scale). The shortened CAS comprises eighteen items of actions by a partner that constitute emotional or physical abuse. For each item, women are asked how frequently a specific behaviour has happened to them during the last twelve months. The scale consists of three dimensions – Emotional Abuse, Physical Abuse and Harassment [31]. During pregnancy and at each follow-up after the birth, two items from the longer version of this scale have been included to identify women who are currently afraid of a partner, or who have ever been afraid of any partner.

Assessment of pre-pregnancy health status is incorporated in the baseline questionnaire (Q1) and telephone interview conducted at 30–32 weeks (CATI 1). Data on socio-demographic characteristics, maternal weight and BMI are being collected in the baseline and follow-up questionnaires.

Information is also being collected on: women's general health and recovery after childbirth; contacts with health services, including primary care and specialist services; physical activity and 'time-out' (when someone else takes care of the baby); practical and emotional support, household composition, partners' involvement in parenting and difficulties in intimate partner relationships; participation in paid work, study and access to paid and unpaid maternity leave.

No urodynamic or other physiological investigations are being undertaken because of the potential negative impact on participation and retention of the cohort, and because the study is focusing on symptom-defined problems.

Pregnancy and birth outcome data will be obtained by medical record review (with women's written consent) according to a detailed written protocol. Women's own accounts of events in pregnancy, labour and birth are being collected in the first two study questionnaires (Q1 & Q2) and telephone interview (CATI 1). Key variables include: method of birth; length of labour; duration of pushing; evidence of obstructed labour; use of epidural, spinal or general anaesthesia; birth position, position of fetus in labour, fetal presentation, degree of perineal and other genital tract trauma, gestation, infant birthweight, and head circumference. Information will also be collected on reproductive history; pregnancy and postnatal complications; and re-attendances at the hospital within six months of the birth. A random 10% sample of medical records will be independently reviewed by two research staff. Final coding will be determined by consensus, with re-checking of the medical record if required. Discrepancies that are not readily able to be resolved in this way will be discussed and resolved at regular team meetings with senior investigators. A record will be kept of all coding discrepancies, and notes taken regarding the rationale for final coding. A further 5% random sample of medical records will be independently re-reviewed to quantify the extent and nature of coding discrepancies.

### Data analysis plan

For aim 1 (incidence, severity and duration of maternal health problems):

▪descriptive frequencies and 95% confidence intervals will be calculated for outcomes of interest at baseline and each follow-up, with stratification to determine the extent of clustering of morbidities and to control for timing of onset of symptoms (prior to pregnancy, during pregnancy, after the birth).

For aim 2 (contribution of pregnancy and birth events to subsequent morbidity):

▪proportions of women reporting outcomes of interest by method of birth (spontaneous vaginal births, forceps assisted vaginal births, vacuum extraction, elective caesarean section, emergency caesarean section) and other obstetric risk factors, including: prolonged first stage labour, fetal position during labour, evidence of obstructed labour in second stage; duration of pushing; fetal presentation at delivery, maternal birth position; use of epidural, spinal or general anaesthesia; degree of perineal trauma; infant birthweight; head circumference; gestation at delivery.

▪multivariable analysis will be undertaken to assess the extent to which method of birth makes an independent contribution to each outcome variable, and to combined morbidity (eg. combined faecal and urinary incontinence). Covariates will include significant labour and birth events, timing of onset of symptoms (pre-pregnancy/during pregnancy/post birth), maternal age and maternal height and early pregnancy BMI. A series of stratified and logistic regression analyses will be performed to validate model choices against stratified data. Generalised linear mixed modelling to take account of repeated measures will be used to determine the effect over time on the associations between health outcomes and covariates.

For aim 3 (reasons for non-disclosure of morbidity to health professionals): descriptive frequencies for prevalent cases will be reported for formal and informal help-seeking and treatment, and for non-disclosure at baseline and each follow-up. Analysis will take into account socio-economic background, the severity and duration of maternal health problems, general health status (SF-36) and extent of co-morbidity.

Assessment of the representativeness of the sample in terms of obstetric and social characteristics will be conducted by comparing study participants with routinely collected Victorian Perinatal Data for births at participating hospitals, and all Victorian births, corresponding with the recruitment period. This will provide an estimate of the representativeness of the sample in terms of exposures of main interest (e.g. method of birth), and covariates (e.g. mothers' age, marital status, country of birth, infant birthweight).

## Discussion

### Changes to the protocol after commencement of recruitment in April 2003

Based on the experience of the pilot phase, it became apparent that our projection that 40% of eligible women would consent to participate on the basis of a mailed invitation was optimistic. A number of additional strategies which aimed to increase participation were implemented from mid 2003 onwards. These included: (i) research team members regularly attending booking clinics at two study hospitals, and 'early-bird' prenatal education classes at a third hospital (which did not have a booking clinic), in order to foreshadow the invitation to take part in the study and answer any questions women had regarding participation; (ii) inclusion of a study leaflet containing a brief outline of the study in the information packages sent to women by participating hospitals prior to their first antenatal visit; (iii) mailing information about the study to general practitioners and specialist obstetricians who provide shared care at participating hospitals and asking them to display materials about the study in waiting areas. We also regularly met with midwives at participating hospitals and produced a six-monthly newsletter to provide information for clinical staff about the study, and ensure that they knew how to contact the research team throughout the period of recruitment. We ruled out adding a second mailed reminder, as we did not want to place women under pressure to participate given the long term nature of follow-up and sensitive nature of some issues covered in questionnaires and interviews.

Our original aim had been to complete recruitment over a 20–24 month period. One of the participating hospitals ceased bookings for maternity care from early 2004. A new hospital within the same network opened in late 2004; we obtained approval to commence recruiting women from this hospital in June 2005. This affected our recruitment to a minor extent. Ultimately recruitment was completed over a two and a half year period, April 2003- December 2005.

### Strengths and limitations of the study

Features of the study which distinguish it from prior research include: the capacity to identify incident cases of morbidity and clustering of health problems; a large enough sample to detect clinically important differences in maternal health outcomes associated with the method of birth; careful exposure measurement involving manual abstraction of data from medical records in order to explore mediating factors and possible causal pathways; and use of a variety of strategies to improve ascertainment of health outcomes such as faecal incontinence, perineal pain and sexual health issues. The latter include use of standardised instruments and questions already tested in prior research with re-piloting to assess acceptability and validity for Victorian women and comparison of results from self-administered questionnaires and telephone interviews.

The rationale for restricting eligibility to women who have reached a minimum of 10 weeks gestation was to limit the number of women enrolling in the study who subsequently experience an early pregnancy loss (spontaneous or induced abortion). Restricting eligibility at enrolment to women who are ≤20 weeks gestation was intended to ensure that data on health status in early pregnancy are available for the whole cohort. A second rationale is to limit the potential for recall bias affecting responses to questions concerning pre-pregnancy health status. A conception cohort would be a more robust design for collection of information on symptoms prior to pregnancy, but in practice is difficult to achieve. The Longitudinal Study of Australian Women (Women's Health Australia) which includes a cohort of younger women recruited at age 18–23 years in 1996, who have now been followed up on two occasions since 1996, has not been designed to facilitate identification of women in the early stages of pregnancy and/or at the time of giving birth [25]. The difficulty and costs of keeping track of a necessarily very large cohort and facilitating follow-up of a sub-sample who become pregnant present major obstacles in designing a study with a capacity to collect information from women immediately preceding and after conception.

The fact that recruitment to the study has been lower than anticipated means that there is increased potential for selection bias related to our primary outcomes. Detailed information on medical conditions, surgical procedures, and symptoms prior to pregnancy has been collected in the baseline questionnaire. Women who report medical conditions, such as Crohn's disease, ulcerative colitis or chronic renal disease that may predispose women to primary outcomes being assessed in the study will be excluded from analyses assessing obstetric risk factors. We will also compare the prevalence of prior medical conditions and symptoms reported by study participants with data from community prevalence studies with respect to urinary and bowel symptoms as a check for evidence of selection bias.

Exclusion of women first attending for pregnancy care at >20 weeks gestation is expected to have a minor impact on the representativeness of the sample. A population-based study conducted by the Victorian Perinatal Data Collection Unit (VPDCU) in 1998 showed that 81.5% of women have their first antenatal visit in the first trimester, and less than 10% attend for a first visit later than 20 weeks [32]. Exclusion of women with insufficient fluency in English to complete written questionnaires and telephone interviews is likely to have greater impact on the representativeness of the sample as approximately 40% of women giving birth at the two tertiary level hospitals during the study period were born overseas in countries where English is not the first language. The restriction of recruitment to women attending public hospitals is a further limitation, especially as it is plausible that differences in the way that care is managed in the private sector (e.g. greater likelihood that labour will be induced and/or augmented, and a higher proportion of women giving birth by caesarean section) may have an impact on maternal health outcomes. Funding constraints and issues of feasibility prevented us from extending the study to these groups.

### Analysis considerations

A major challenge in planning analyses assessing causal pathways is the extent to which obstetric risk factors are linked, and the associated difficulty in analysis of sorting out the impact of method of birth (as the 'exposure of main interest') from other exposure and treatment effects. Recently there has been much debate about the possible benefits of elective caesarean section in protecting women's pelvic floor and preventing urinary incontinence [9-12]. Although sample size calculations for the study were not based on comparisons of spontaneous vaginal birth with elective caesarean section, the study will have adequate power for this comparison. Additional file 1 (table 2) and 2 (table 3) provide a summary of pregnancy and birth cohort studies which have assessed the role of obstetric factors for urinary incontinence in pregnancy and after childbirth. Most of the included studies with data on operative births report that caesarean section is protective against urinary incontinence after childbirth compared with vaginal birth [see Additional File 1 and Additional File 2]. However, few of these studies incorporate comprehensive assessment of obstetric risk factors. Only half of the included studies obtained information on obstetric events by reviewing medical case notes [34-36,38,42,45,47,49,50,52-54], and two of these studies relied in part on information obtained from computerised records [53,54]. Many studies do not differentiate women who have an emergency caesarean section in labour from those having an elective caesarean section prior to commencement of labour [36,38-40,46,48,51-54]. Less than half include measures of fetal or infant size [34-36,45,47,48,53,54], maternal size (height, pre-pregnancy BMI) [34,36,48,50,52-54], or genital tract trauma [34,45,47-49,52-54], and only a handful of studies include data on use of analgesia and anaesthesia [43,50,52-54], fetal position in labour [35,39,50] or maternal position in second stage [53]. In contrast, duration of second stage labour is commonly collected as a proxy variable for obstructed labour [33-36,39-41,43,48-51,53,54]. Inconsistencies in the way information on length of labour is recorded, and lack of data on other indicators of possible obstructed labour (e.g. position of the fetus, descent of the fetal head) make it difficult to interpret this data.

In summary, the striking features of this literature are: the lack of consistency in the approaches to data collection taken by different research groups, and the limited extent to which variables related to events in labour other than method of birth have been taken into consideration. Many studies included in Tables 2 and 3 also have inadequate study power for examining obstetric risk factors [33-35,40,43-45,48-50].

Two recently published papers by Glazener et al [53] and MacArthur et al [54] drawing on data from a large multi-centre prospective birth cohort are a welcome addition to this literature. Both papers report carefully conducted analyses for incident cases of morbidity considering a broader range of obstetric and maternal risk factors, including maternal age and BMI. Glazener and colleagues focus on primiparous women at three months postpartum distinguishing between women whose incontinence commenced in pregnancy and those who developed symptoms after giving birth to their first child, while MacArthur et al report on persistent incontinence six years after the index birth adjusting for first delivery mode, maternal age at first birth, and parity. One limitation of this study is that identification of incident cases (onset of symptoms in pregnancy or after birth) is reliant on maternal recall at three months postpartum.

In the Maternal Health Study the baseline survey completed in early pregnancy includes detailed questions regarding urinary and other symptoms: (i) 'at any stage in your life before the current pregnancy' and (ii) in the twelve months prior to the pregnancy. We have also asked separately about childhood enuresis and relevant medical conditions and prior surgery. Detailed information on a comprehensive list of obstetric covariates is being collected from hospital medical records and will be supplemented by women's own accounts of events in labour and birth collected in the questionnaire completed at three months postpartum (Q2). This will enable us to look at variables that are poorly recorded or not available in medical records. For example, we have included questions in Q2 regarding maternal position and method of pushing in active second stage labour. We are also endeavouring to collect information from medical records regarding the timing of events in labour, for example, time of administration of epidural or spinal analgesia/anaesthesia and time that women commenced pushing in second stage labour. Precise definitions have been developed and pilot tested for all obstetric data items being collected from medical records and Q2.

The first step in analysis will be to assess data quality to identify variables that are poorly or inconsistently recorded in medical records and/or women's accounts of labour and birth events. One aspect of this will be to assess the extent of agreement between data collected in self-administered questionnaires and information abstracted from hospital medical records for pregnancy and birth events.

**Figure 2:**
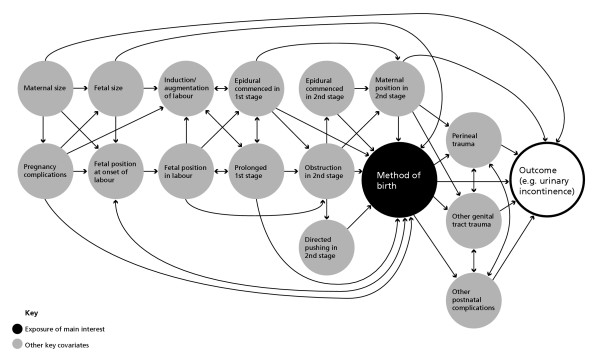
**Association chain graph for postnatal maternal health outcomes **● Exposure of main interest ● Other key covariates

Figure 2 provides a schematic overview of key covariates that will be considered in analysis. The arrows represent possible causal associations between covariates based on available epidemiological evidence regarding obstetric risk factors for maternal morbidity. Planning analyses using causal acyclic diagrams has been advocated by Greenland and others as a useful strategy for identifying variables that may mediate or moderate the relationship between an exposure of main interest and primary health outcome/s [55,56]. Figure 2 illustrates the complexity of possible causal pathways and relationships between obstetric risk factors for maternal morbidity. Considering our hypothesis that vaginal birth assisted by forceps or vacuum extraction is an 'independent' risk factor for urinary incontinence, the diagram illustrates the multiple covariates and combinations of covariates preceding the delivery and subsequent to delivery that may mediate the relationship between the mode of birth and the outcome of urinary incontinence. To take one example, the estimated size of the fetus as large for gestational age might lead directly to the decision to conduct an elective caesarean section, or alternatively, via a range of possible pathways to obstruction in second stage labour and a vaginal birth assisted by forceps or vacuum extraction or an emergency caesarean section. A number of other factors may influence the outcome, including the position of the fetus in second stage labour, length of time in active second stage, whether or not an episiotomy is cut and the extent of other genital tract trauma. The conventional statistical approach – used by many researchers – of adjusting for those covariates such as infant size for which data are available is likely to lead to erroneous conclusions. This is not only because data are rarely available for the full range of covariates illustrated in Figure 2, but also because such analyses fail to consider the complex range of possible pathways involved. Simple adjustment for fetal size assumes that the impact will be the same irrespective of other aspects of labour and birth. Biologically this seems somewhat implausible given the extra pressure on the pelvic floor associated with a long labour and large baby often in occipito-posterior position. While these other factors can also be adjusted for in analysis, it may be more useful to conduct separate analyses restricted to women who do and do not experience labour. This is likely to yield less biased estimates of effect for specific causal pathways [57]. The timing of onset of symptoms (and symptom resolution) presents further challenges in analysis. Separate stratified analyses will be performed for incident and prevalent cases of morbidity.

## Abbreviations

BMI Body mass index

CAS Composite abuse scale

CATI Computer assisted telephone interview

CS Caesarean section

EPDS Edinburgh Postnatal Depression Scale

MCHR Mother & Child Health Research

NICU Neonatal intensive care unit

OP Occipito-posterior

PP Postpartum

Q1 Questionnaire 1

SF-36 Short Form 36, Health status measure

SVB Spontaneous vaginal birth

UI Urinary incontinence

USI Urinary stress incontinence

UUI Urinary urge incontinence

VE Vacuum extraction

## Competing interests

The author(s) declare that they have no competing interests.

## Authors' contributions

SB conceived the overall study design and wrote the successful grant application with advice on the design from JL and statistical advice from Lyn Watson. EMcD developed the data collection procedures with participating hospitals with input from SB and Creina Mitchell. SB, JL, EMcD and AK were involved in the development and piloting of study instruments with input from Chris Bessell, Shaun Brennecke, Robert Burrows, Jane Gunn, Creina Mitchell, Lyn Watson and Peter Wein. SB, AK and JL developed the data abstraction form and protocol for abstracting information from medical records with input from Maggie Flood, Leisje Toomey, Martine Kilby, EMcD, Chris Bessell, Robert Burrows, Jane Gunn and Peter Wein. AK developed the protocol for conducting telephone interviews and training program for interviewers with input from SB, JL, EMcD and Jane Gunn. EMcD is responsible for data management, coding of self-administered questionnaires and data cleaning. AK is responsible for co-ordinating the conduct of telephone interviews and management of CATI data. All the authors have commented on drafts of this protocol. SB is responsible for the overall direction of the project and wrote the protocol using original documents and records.

## Pre-publication history

The pre-publication history for this paper can be accessed here:



## Supplementary Material

Additional File 1 **Table 2 Pregnancy cohort studies assessing role of obstetric risk factors for urinary incontinence in pregnancy and after childbirth**. The table provides a structured summary of study design, methods, sample, outcome measures, exposure measures and analyses for pregnancy cohort studies assessing obstetric risk factors. Click here for file

Additional File 2 **Table 3 Birth cohort studies assessing role of obstetric risk factors for urinary incontinence in pregnancy and after childbirth. **The table provides a structured summary of study design, methods, sample, outcome measures, exposure measures and analyses for birth cohort studies assessing obstetric risk factors. Click here for file

## References

[B1] MacArthur C, Lewis M, Knox E (1991). Health After Childbirth. London: Her Majesty's Stationery Office.

[B2] Glazener C, Abdall M, Stroud P, Naji S, Templeton A, Russell I (1995). Postnatal maternal morbidity: extent, causes, prevention and treatment. Br J Obstet Gynaecol.

[B3] Brown S, Lumley J (1998). Maternal health after childbirth: results of an Australian population based survey. British Journal of Obstetrics and Gynaecology.

[B4] Glazener C (1997). Sexual function after childbirth: women's experiences, persistent morbidity and lack of professional recognition. Br J Obstet Gynaecol.

[B5] Barrett G, Pendry E, Peacock J, Victor C, Thakar R, Manyonda I (2000). Women's sexual health after childbirth. BJOG.

[B6] Campbell J (2002). Health consequences of intimate partner violence. Lancet.

[B7] Bowen E, Heron J, Waylen A, Wolke D, ALSPAC Study Team (2005). Domestic violence risk during and after pregnancy: findings from a British longitudinal study. BJOG.

[B8] Bacchus L, Mezey G, Bewley S (2004). Domestic violence: prevalence in pregnant women and associations with physical and psychological health. Eur J Obstet Gynecol Reprod.

[B9] Sultan A, Stanton S (1996). Preserving the pelvic floor and perineum during childbirth – elective caesarean section? (commentary). Br J Obstet Gyn.

[B10] Al-Mufti R, McCarthy A, Fisk N (1996). Obstetricians' personal choice and mode of delivery (letter). Lancet.

[B11] Farrell S (2002). Caesarean section versus forceps-assisted vaginal birth: It's time to include pelvic injury in the risk-benefit equation (commentary). CMAJ.

[B12] Minkoff H, Chervenak F (2003). Elective primary caesarean delivery. New Engl J Med.

[B13] MacLennan A, Taylor A, Wilson D, Wilson D (2000). The prevalence of pelvic floor disorders and their relationship to gender, age, parity and model of delivery. Br J Obstet Gynaecol.

[B14] Rortweit G, Daltveit A, Hannstad Y, Hunskaar S (2003). Urinary incontinence after vaginal delivery or caesarean section. New Engl J Med.

[B15] MacArthur C, Glazener C, Lancashire R, Herbison P, Wilson D, Grant A (2005). Faecal incontinence and mode of first and subsequent delivery. BJOG.

[B16] Dean AG, Dean JA, Burton AH, Dicker RC Epi Info: word processing, database and statistics program for epidemiology on microcomputers.

[B17] Gunthorpe W, Brown W, Redman S (2000). The development and evaluation of an incontinence screening questionnaire for female primary care. Neurourol Urodyn.

[B18] Sandvik H, Seim A, Vanvik A, Hunskaar S (2000). A severity index for epidemiological surveys of female urinary incontinence: comparison with 48-hour pad-weighing tests. Neurourol Urodyn.

[B19] Sandvik H, Hunskaar S, Seim A, Hermstad R, Vanvik A, Bratt H (1993). Validation of a severity index in female urinary incontinence and its implementation in an epidemiological survey. J Epid Comm Health.

[B20] Hanley J, Capewell A, Hagen S (2001). Validity study of the severity index, a simple measure of urinary incontinence in women. BMJ.

[B21] Talley N, Boyce P, Owen B (1995). Initial validation of a bowel symptom questionnaire and measurement of chronic gastrointestinal symptoms in Australians. Aust NZ J Med.

[B22] Kalantar J, Howell S, Talley N (2002). Prevalence of faecal incontinence and associated risk factors. An underdiagnosed problem in the Australian community?. Med J Aust.

[B23] Lam T, Kennedy M, Chen F, Lubowski D, Talley N (1999). Prevalence of faecal incontinence: obstetric and constipation-related factors; a population-based study. Colorectal Disease.

[B24] Richters J, Grulich A, de Visser R, Smith A, Rissell C (2003). Sex in Australia : sexual and emotional satisfaction in regular relationships and preferred frequency of sex among a representative sample of adults. Aust NZ J Public Health.

[B25] Brown W, Dobson A, Bryson L, Byles J (1999). Women's Health Australia: on the progress of the main cohort studies. J Women's Health Gend Based Med.

[B26] Melzak P (1975). The McGill pain questionnaire: major properties and scoring methods. Pain.

[B27] Cleeland CS, Ryan KM (1994). Pain assessment: global use of the Brief Pain Inventory. Ann Acad Med Singapore.

[B28] Jenkinson C, Coulter A, Wright L (1993). Short form 36 (SF-36) health survey questionnaire: normative data for adults of working age. BMJ.

[B29] Murray L, Carothers AD (1990). The validation of the Edinburgh Postnatal Depression Scale on a community sample. Br J Psychiatry.

[B30] Boyce P, Stubbs R, Todd A (1993). The Edinburgh Postnatal Depression Scale: validation in an Australian sample. Aust NZ J Psychiatry.

[B31] Hegarty KL, Sheehan M, Schonfeld C (1999). A multidimensional definition of partner abuse: Development and preliminary validation of the Composite Abuse Scale. J Family Violence.

[B32] Halliday J, Ellis I, Stone C (1999). *WUDWAW Report on models of antenatal care*. Melbourne: VPDCU.

[B33] Dimpfl T, Hesse U, Schussler B (1992). Incidence and cause of postpartum urinary stress incontinence. Eur J Obstet Gyn Reprod Biol.

[B34] Chaliha C, Kalia V, Stanton S, Sultan A (1999). Antenatal prediction of postpartum urinary and faecal incontinence. Obstet Gynecol.

[B35] Farrell, Allen V, Baskett T (2001). Parturition and urinary incontinence in primiparas. Obstet Gynecol.

[B36] Eason E, Labrecque M, Marcoux S, Mondor M (2004). Effects of carrying a pregnancy and of method of delivery on urinary incontinence: a prospective cohort study. BMC Pregnancy Childbirth.

[B37] Schytt E, Lindmark G, Waldenstrom U (2004). Symptoms of stress incontinence1 year after childbirth: prevalence and predictors in a national Swedish sample. Acta Obstet Gynecol Scand.

[B38] Klein M, Kaczorowski J, Firoz T, Hubinette M, Jorgensen S, Gauthier R (2005). A comparison of urinary and sexual outcomes in women experiencing vaginal and caesarean births. J Obstet Gynaecol Can.

[B39] Iosif S (1981). Stress incontinence during pregnancy and puerperium. Int J Gynaecol Obstet.

[B40] Viktrup L, Lose G, Rolff M, Barfoed K (1992). The symptom of stress incontinence caused by pregnancy or delivery in primiparas. Obstet Gynecol.

[B41] MacArthur C, Lewis M, Bick D (1993). Stress incontinence after childbirth. Br J Midwifery.

[B42] Wilson P, Herbison R, Herbison G (1996). Obstetric practice and the prevalence of urinary incontinence three months after delivery. Br J Obstet Gynaecol.

[B43] Krue S, Jensen H, Agger A, Rasmussen K (1997). The influence of infant birth weight on postpartum stress incontinence in obese women. Arch Gynecol Obstet.

[B44] Groutz A, Gordon D, Keidar R, Lessing J, Wolman I, David M, Chen B (1999). Stress urinary incontinence: prevalence among nulliparous compared with grand multiparous premenopausal women. Neurol Urodynamics.

[B45] Arya L, Jackson N, Myers D, Verma A (2001). Risk of new-onset urinary incontinence after forceps and vacuum delivery in primiparous women. Am J Obstet Gynecol.

[B46] Thompson J, Roberts C, Currie M, Ellwood D (2002). Prevalence and persistence of health problems after childbirth: associations with parity and method of birth. Birth.

[B47] Fenner D, Genberg B, Brahma P, Marek L, DeLancey L (2003). Fecal and urinary incontinence after vaginal delivery with anal sphincter disruption in an obstetrics unit in the United States. Am J Obstet Gynecol.

[B48] Burgio K, Zyczynski H, Locher J, Richter H, Reden D, Wright K (2003). Urinary incontinence in the 12-month postpartum period. Obstet Gynecol.

[B49] Yip S-K, Sahota D, Chang A, Chung T (2003). Effects of one interval vaginal delivery on the prevalence of stress urinary incontinence: a prospective cohort study. Neurol Urodynamics.

[B50] Liebling R, Swingler R, Patel R, Verity L, Soothill P, Murphy D (2004). Pelvic floor morbidity up to one year after difficult instrumental delivery and caesarean section in the second stage of labor: a cohort study. Am J Obstet Gynecol.

[B51] Casey B, Schaffer J, Bloom S, Heartwell S, McIntire D, Leveno K (2005). Obstetric antecedents for postpartum pelvic floor dysfunction. Am J Obstet Gynecol.

[B52] Ewings P, Spencer S, Marsh H, O'Sullivan M (2005). Obstetric risk factors for urinary incontinence and preventative pelvic floor exercises: cohort study and nested randomized controlled trial. J Obstet Gynaecol.

[B53] Glazener CMA, Herbison GP, MacArthur C, Lancashire R, McGee MA, Grant A, Wilson PD (2006). New postnatal urinary incontinence: obstetric and other risk factors in primiparae. BJOG.

[B54] MacArthur C, Glazener CMA, Wilson DP, Lancashire R, Herbison GP, Grant A (2006). Persistent urinary incontinence and delivery mode history: a six-year longitudinal study. BJOG.

[B55] Greenland S, Pearl J, Robins J (1999). Causal diagrams for epidemiologic research. Epidemiology.

[B56] Greenland S, Brumback B (2002). An overview of relations among causal modelling methods. Int J Epi.

[B57] Christenfeld N, Sloan R, Carroll D, Greenland S (2004). Risk factors, confounding and the illusion of statistical control. Psychosomatic Medicine.

