# Supplementary material for: Maternal health study: a prospective cohort study of nulliparous women recruited in early pregnancy
Source: BMC Pregnancy Childbirth. 2006 Apr 11;6:12. doi: 10.1186/1471-2393-6-12 (PMC1463006; doi:10.1186/1471-2393-6-12)
Supplement: Additional File 1 — Table 2 Pregnancy cohort studies assessing role of obstetric risk factors for urinary incontinence in pregnancy and after childbirth. The table provides a structured summary of study design, methods, sample, outcome measures, exposure measures and analyses for pregnancy cohort studies assessing obstetric risk factors. [file 1471-2393-6-12-S1.doc]

**Table 2: Pregnancy cohort studies assessing role of obstetric risk factors for urinary incontinence in pregnancy and after childbirth**

| **Authors** | **Type of study** | **Sample** | **Outcome measure/s** | **Exposure measures** | **Analysis** |
| --- | --- | --- | --- | --- | --- |
| Dimpfl et al,  European J Obstet Gynaecol, 1992 [33] | Prospective pregnancy cohort with follow-up at 6 and 12 weeks pp, no information regarding source of data on birth events | n=350  Recruited in 3^rd^ trimester, incl. primips and multips, women with incontinence before pregnancy and women with concomitant urge incontinence excluded  Setting: teaching hospital, Munich, 1986 | Urinary stress incontinence according to International Continence Society definition  53.5% had USI in pregnancy  6.2% had USI at 6 weeks  6.2% had USI at 12 weeks  3.7% onset of USI after birth | Parity  Method of birth (SVB, forceps, VE, CS)  Length of labour  Epidural vs pudendal analgesia  Episiotomy  Perineal trauma  Infant birthweight  Head circumference | Univariate associations for all exposure variables  - CS protective (0% of women with USI at 6 weeks, cf 10.1% for forceps, 9.1% for VE, and 6.1% for SVB)  - pudendal analgesia associated with USI (9.1% cf 3.1% for epiduaral)  - no significant associations with other obstetric risk factors |
| Chaliha et al,  Obstet Gynecol, 1999 [34] | Prospective pregnancy cohort with follow-up interviews at 12 weeks pp, data on birth events abstracted from case notes | n=549  Recruited after 34 weeks gestation, nulliparous women with no history of recurrent urinary tract infection urinary tract abnormality, anorectal surgery or trauma, and no active urinary tract or bowel infection; participants representative of hospital population;  100% retention at 3 months pp  Setting: maternity unit, London, UK, 1996-1997. | Urinary stress incontinence & urinary urge incontinence using standardised questionnaire  3.1% had USI before pregnancy  35.7% had USI in late pregnancy  12.4% had USI at 3 months pp  0.5% had UUI before pregnancy  8.0% had UUI in late pregnancy  2.2% had UUI at 3 months pp  5.5% (30/549) had onset of UI after birth | Method of birth (SVB, instrumental, CS)  Augmentation  Epidural  Length of labour  Perineal trauma  Fetal weight  Fetal head circumference  BMI  Smoking  Family history of incontinence  Markers of collagen weakness | Univariate associations for all exposure variables  Stratified analysis comparing method of birth groups controlling for timing of onset of symptoms:  - for incident cases (onset of symptoms after birth) no significant associations with obstetric risk factors  - 13.1% of women who had SVB had *de novo* USI at 3 months pp, cf 15.3% for forceps/VE, and 8.4% for CS  - 2.0% of women who had SVB had *de novo* urge incontinence at 3 months pp, cf 4.0% for forceps/VE and 0.7% for CS |
| Farrell et al,  Obstetrics and Gynecology, 2001 [35] | Prospective pregnancy cohort with follow-up at 6 weeks and 6 months pp, with data on birth events abstracted from hospital medical record | n=690  Recruited at prenatal visits or prenatal classes, nulliparous women, with no history of urinary tract abnormalities or significant medical illness, excluding stillbirths & neonatal deaths; 81% retention at 6 weeks pp & 70% at 6 months pp  Setting: Teaching hospital, Nova Scotia, Canada, 1996-98 | Urinary incontinence: ‘accidental loss of urine’  26% report UI at 6 months pp  13.4% had symptoms UI prior to pregnancy | Method of birth (SVB, forceps, CS)  Length of active 2^nd^ stage  Fetal position at delivery  Analgesia  Infant birthweight  Head circumference | Univariate associations for all exposure measures  - CS protective  - no association with other exposure measures  Stratified analysis comparing method of birth groups controlling for prior symptoms of UI  - CS protective  - forceps associated with increased odds compared with CS and SVB: OR = 3.1(1.7-5.9), OR=1.5(1.0-2.30) respectively  Multivariate analysis using logistic regression adjusting for length of 2^nd^ stage and duration of epidural  - no difference in UI at 6 months pp associated with forceps (adj OR = 1.3 [0.6-2.5]) |
| Eason et al  BMC Pregnancy and Childbirth, 2004 [36] | Prospective pregnancy cohort nested in RCT of perineal massage with follow-up at 3 months pp, data on birth events abstracted from case notes and detailed data on perineal trauma completed by attending medical staff | n=949  Recruited at 30-35 wks gestation, primiparous & multiparous women; 79% retention at 3 months pp  Setting: Teaching hospital, Quebec, Canada, 1994-95 | Urinary stress incontinence: involuntary loss of urine when cough, laugh or sneeze ‘on at least 2 occasions’  22.3% had USI before pregnancy  65.1% had USI in 3^rd^ trimester  31.1% had USI at 3 months pp | Parity  Method of birth (SVB, forceps, VE, CS)  Length of labour  Epidural analgesia  Infant birthweight  Episiotomy  Perineal trauma  BMI  Weight gain in pregnancy  Maternal age | Univariate associations for all exposure measures  - no association with episiotomy, epidural, duration 2^nd^ stage, infant birthweight  Multivariate analysis using logistic regression to adjust for previous vaginal births, method of birth, maternal age, duration 2^nd^ stage, episiotomy, timing of onset of USI, BMI  - no difference in USI at 3 months pp for SVB vs forceps or VE  - CS protective for USI at 3 months pp cf SVB (adj OR= 0.27 [0.1-0.5]) |
| Schytt et al,  Acta Obstet Gynecol Scand, 2004 [37] | Prospective pregnancy cohort with follow-up at 8 weeks and 12 months pp, data on birth events obtained from Swedish birth register | n=3061  Recruited in early pregnancy (mean 16 weeks), primips and multips; 80% retention at 12 m pp (2450 women completed all stages of follow-up)  Setting: Sweden, population-based sample, 1999-2000 | Urinary stress incontinence: loss of urine during physical exertion (e.g. sneezing, jumping)  In primips (n=1051):  30.5% had USI in 3^rd^ trimester  43.5% at 4-8 weeks pp  18.4% at 12 months pp | Parity  Method of birth (SVB, forceps/VE, emergency CS and elective CS)  Prior symptoms of USI  Fetal position  Birthweight  Head circumference  Perineal trauma  BMI  Constipation | Univariate associations for all exposures  Stratified analyses controlling for parity  - CS protective for USI in primiparous women  - other obstetric factors that raised risk of USI: OP position, large head circumference, perineal tears (all borderline associations)  Multivariate analysis incorporating prior symptoms (in pregnancy and/or 4-8 weeks pp) found no significant associations with obstetric factors |
| Klein et al,  JOGC, 2005 [38] | Prospective pregnancy cohort nested in RCT of routine vs selective episiotomy with follow-up at 3 months pp, data on birth events abstracted from case notes | n=999  Recruited at 34-36 weeks gestation, includes primiparous and multiparous women at low risk and expecting vaginal birth; 95.7% retention at 3 months pp  Setting: 3 teaching hospitals, Montreal, Canada 1990-1991 | Unspecified urinary incontinence: ‘trouble with loss of urine’ in any circumstances  Urinary stress incontinence: ‘trouble with loss of urine when you laugh, cough or sneeze’  In primips:  30.2% had USI at 3 months pp | Parity  Method of birth (vaginal births, CS [majority emergency CS])  Prior symptoms of UI | Univariate associations for primiparous women with method of birth  - CS protective for USI in primiparous women (12.8% vs 34.5%)  Stratified analysis comparing primiparous women who had vaginal birth with those having CS controlling for history of USI prior to pregnancy:  - CS protective among women with no prior symptoms (OR=0.25[0.01-0.64]) |

**Notes**: CS caesarean section; SVB spontaneous vaginal birth; VE vacuum extraction; UI unspecified urinary incontinence; USI urinary stress incontinence; UUI urinary urge incontinence; pp postpartum; OP occipito-posterior; BMI body mass index
