# Supplementary material for: Maternal health study: a prospective cohort study of nulliparous women recruited in early pregnancy
Source: BMC Pregnancy Childbirth. 2006 Apr 11;6:12. doi: 10.1186/1471-2393-6-12 (PMC1463006; doi:10.1186/1471-2393-6-12)
Supplement: Additional File 2 — Table 3 Birth cohort studies assessing role of obstetric risk factors for urinary incontinence in pregnancy and after childbirth. The table provides a structured summary of study design, methods, sample, outcome measures, exposure measures and analyses for birth cohort studies assessing obstetric risk factors. [file 1471-2393-6-12-S2.doc]

**Table 3: Birth cohort studies assessing role of obstetric risk factors for urinary incontinence in pregnancy and after childbirth**

| **Authors** | **Type of study** | **Sample** | **Outcome measure/s** | **Exposure measures** | **Analysis** |
| --- | --- | --- | --- | --- | --- |
| Iosif et al,  Int J Gyn Obstet  1981 [39] | Prospective birth cohort with follow-up at 6-12 months pp of women who reported USI in 1^st^ study questionnaire, no information on source of data on obstetric events | n=1411  Recruited on postnatal ward (7-14d pp); primiparous and multiparous women; 94% response fraction to baseline questionnaire, 95% of eligible women (those reporting USI at baseline) responded to 2^nd^ questionnaire  Setting: Women’s clinic, Lund, Sweden, 1970s | Urinary stress incontinence: involuntary loss of urine when coughing, lifting, climbing stairs  22% of women reported USI during pregnancy and/or postpartum  1.8% had USI before pregnancy  15.7% had USI during pregnancy  4.1% had USI at 7-14 d pp (>half of these women remained incontinent at 6-12 months pp) | Method of birth (SVB, forceps, CS)  Length of labour  Deflection of fetal head  Infant birthweight | Univariate associations with obstetric factors restricted to women who developed symptoms in pregnancy or after birth  - no significant associations found |
| Viktrup et al  Obstetrics and Gynecology, 1992 [40] | Prospective birth cohort followed up at 3 months pp, with further follow-up at 12 months pp for women who reported USI at 3 months pp, no information on source of data on obstetric events (possibly case notes) | n=305  Recruited on postnatal ward (3-5d pp); primiparous women only; no information on response fraction  Setting: single maternity unit, Copenhagen, Denmark | Urinary stress incontinence: leakage of urine associated with physical stress  Urge incontinence; leakage accompanied by strong desire to void  4% had USI before pregnancy  29% developed USI during pregnancy (of these women 3% had symptoms at 3 months pp, and 1% at 12 months pp)  7% onset of USI after birth (of these women 2% had symptoms at 3months, and 2% at 12 months) | Method of birth (SVB, forceps/VE, CS)  Length of labour  Episiotomy  Infant birthweight  Head circumference | Univariate associations with all exposure methods  Stratified analyses controlling for timing of onset of USI (before pregnancy, during pregnancy, after birth):  - onset of USI after birth associated with longer labour, no associations with episiotomy, infant birthweight or head circumference  - CS protective for women with no symptoms prior to birth (0% vs 13% reported *de novo* USI after birth) |
| MacArthur et al,  Br J Mid, 1993 [41], Health After Childbirth, HMSO, 1991 [1] | Retrospective single hospital birth cohort, cross sectional postal survey sent Jan 1987 to women who gave birth to most recent child between 1978-1985 (up to 11 years post index birth), data on birth events taken from computerised case notes | n=11,701  Primiparous and multiparous women; response fraction of 30%  Setting: teaching hospital, Birmingham, UK, 1978-1985 | Urinary stress incontinence: ‘hard to hold urine when jump, sneeze etc.’  15.2% reported new symptoms USI within 3 months of birth  5.4% had chronic or recurring symptoms predating index birth | Parity  Method of birth (SVB, rotational forceps, plain forceps, elective CS, emergency CS)  Onset of labour  Duration 1^st^ & 2^nd^ stage  Fetal presentation  Episiotomy  Perineal laceration  Postpartum haemorrhage  Gestation  Maternal height  Infant birthweight  Infant head circumference  Infant length | Univariate associations for all exposure measures with outcome of new symptoms USI within 3 months of index birth  - USI associated with: longer 1^st^ & 2^nd^ stage labour, epidural, OP presentation, forceps, episiotomy, higher birthweight infants, large head circumference, longer babies, perineal lacerations  Multivariate analysis using discriminant analysis adjusting for all factors found to be significantly associated with USI at univariate level:  - main predictors of USI: higher maternal age, longer 2^nd^ stage, higher birthweight (>3700g); forceps and perineal lacerations predictive when duration of 2^nd^ stage labour left out of model  - CS protective |
| Wilson et al,  BJOG, 1996 [42] | Birth cohort, cross sectional survey at 3 months pp, data on birth events abstracted from case notes | n =1505  Primiparous and multiparous women; 70.5% response fraction  Setting: Single maternity unit, Dunedin, NZ 1989-91 | Urinary stress incontinence: ‘leakage with coughing, laughing or sneezing’  Urinary urge incontinence: not defined  Any incontinence =  34.3% total sample  29.7% primiparous women  21.4% primiparous women with onset symptoms >12 weeks gestation | Parity  Method of birth (SVB, Forceps, CS)  BMI  Pelvic floor exercises | Univariate associations for all exposure measures  Stratified analysis comparing all method of delivery groups controlling individually for parity and onset of symptoms >12 weeks gestation  Multivariate analysis using logistic regression to adjust for method of birth, parity, BMI and pelvic floor exercises in pregnancy:  - no difference in outcome for SVB vs forceps  - CS protective cf SVB (adjOR = 0.4[0.2-0.7]) |
| Krue et al,  Arch Gynecol Obstet, 1997 [43] | Birth cohort, cross sectional survey at 6-30 months pp, no information on source of data on obstetric events | n=119  Primiparous and multiparous women with pre-pregnancy BMI of ≥30, excluding women who had a CS in index pregnancy or a subsequent birth; no information on response fraction  Setting: teaching hospital, Denmark, 1993-99 | Urinary stress incontinence  Urge incontinence  Mixed incontinence  - at least once per week (no information regarding measures)  Prior to pregnancy:  8% had USI  1.6% had UUI  10% had mixed incontinence  Post birth:  31.9% had USI  5% had UUI  12.6% had mixed incontinence | Method of birth (SVB, forceps/VE)  Infant birthweight  Pudendal anaesthesia | Univariate associations for all exposure measures  Stratified analysis assessing contribution of infant birthweight controlling for onset of symptoms (prior to pregnancy/during pregnancy/after birth)  - no significant association between infant birthweight and USI |
| Brown et al,  BJOG, 1998 [3] | Population-based birth cohort, cross sectional survey at 6-7 months pp, data on obstetric events obtained from questionnaire responses | n=1336  Primiparous and multiparous women, excluding those who had a stillbirth or neonatal death; 62.5% response fraction  Setting: State of Victoria, Australia, 1993-94 | Urinary incontinence: ‘loss of bladder control a problem in the first 6 months pp  10.9% total sample reported UI  10.4% primiparous women reported UI | Parity  Method of birth (SVB, forceps, VE, elective CS, emergency CS)  Length of labour  Infant birthweight  Perineal trauma | Univariate associations for all exposure measures  Stratified analyses comparing assisted vaginal births with SVB controlling individually for duration of labour (<6/≥12hrs), infant birthweight (<4000/≥4000g), and perineal trauma weighted for parity  - assisted vaginal births had higher likelihood of UI, controlling for duration of labour (wtOR=1.46[0.9-2.4]); higher infant birthweight (wtOR=1.90[1.2-3.1]), & perineal trama (wtOR=1.45[0.9-2.6]) |
| Groutz et al,  Neurology and Urodynamics,  1999 [44] | Birth cohort recruited on postnatal ward, cross sectional interview study at 2-3 days pp, no information on source of data on obstetric events | n=300  Consecutive sampling to obtain 3 groups of women meeting inclusion criteria:  (i) nulliparous women (n=100)  (ii) primiparous women with 1 previous vaginal birth (n=100)  (iii) grand multiparous women with at least 5 previous vaginal births(n=100)  Setting: single maternity unit, Tel Aviv, Israel, 1997 | Urinary stress incontinence: defined as involuntary loss of urine when laughing, coughing, sneezing etc.  28% of nulliparous women had USI in pregnancy, cf 50% of parous women  5% of nulliparous women had persistent USI after birth, cf 11% of primiparous women &  21 % of grand multiparous women | Parity  Mode of birth in index pregnancy (SVB, instrumental)  Mode of birth in previous pregnancy (SVB, instrumental)  Infant birthweight  Maternal age | Univariate associations reported for outcome of persistent USI (ie at 2-3 days pp) with mode of 1^st^ birth, and infant birthweight  - no significant association with infant birthweight, or mode of 1^st^ birth comparing SVB and instrumental vaginal births |
| Arya et al,  Am J Obstet Gynecol, 2001 [45] | Prospective birth cohort with follow-up at 2 weeks, 3 months and 12 months pp, data on obstetric events obtained from hospital case notes | n=315  Consecutive primiparous women who gave birth vaginally, excluding women who had a history of urinary incontinence before or during pregnancy, women with diabetes or known neurological disease  Setting: Single maternity unit, Florida, United States, 1999 | Urinary stress incontinence: defined as leakage provoked by physical stress, coughing etc  Urge incontinence: leakage accompanied by strong desire to void (instrument designed by Viktrup, 1992)  13.3% had USI at 2 weeks  6.6% had USI at 3 months  9.2% had USI at 12 months | Method of birth (SVB, forceps, VE)  Maternal age  Duration of 1^st^ & 2^nd^ stage labour  Anterior & posterior ‘vaginal lacerations’  Infant birthweight | Univariate associations for method of birth  Multivariate analysis using Cox proportional regression for outcome of developing USI over first 12 months pp adjusting for method of birth, age, infant birthweight, duration of 1^st^ & 2^nd^ stage labour:  - forceps increased risk (adj RR = 3.5[1.1-11.4])  - no association with other covariates |
| Thompson et al,  Birth, 2002 [46] | Population-based prospective birth cohort recruited on postnatal wards, with follow-up at 8, 16 and 24 weeks pp, data on obstetric events obtained from questionnaire responses | n=1295  Primiparous and multiparous women;  70% response fraction to baseline survey, 92% retention at 24 wks pp  Setting: Australian Capital Territory, Australia 1997 | Urinary stress incontinence: ‘hard to hold urine when coughing, sneezing or exercising’  19% of women had USI at 8 weeks  11% of women had USI at 17-24 weeks | Parity  Method of birth (SVB, forceps/VE, CS)  Length of labour | Univariate associations for method of birth and parity  - no difference in USI for SVB vs forceps/VE  - CS protective for USI at 1-8 weeks (OR=0.25[0.14-0.44] cf assisted vaginal births, but difference not statistically significant at 16 weeks and 24 weeks  Multivariate analyses not reported for outcome of USI |
| Fenner et al,  Am J Obstet Gynecol  2003 [47] | Birth cohort, cross sectional survey at 6 months pp, with data on birth events abstracted from hospital medical records | n=943  Primiparous women who gave birth vaginally, with no prior symptoms of urinary incontinence before pregnancy, no significant medical illness or medication that could affect urinary or bowel function, no urinary tract abnormalities; 32% response fraction for urinary symptom questionnaire; authors note selection bias towards women with 3^rd^/4^th^ degree tears.  Setting: teaching hospital, Michigan, US 1997-2000 | Urinary stress incontinence: ‘wet when cough, laugh or sneeze’  Urge incontinence: ‘leak urine if hear running water, not able to wait to use toilet’  21.3% had USI at 6 months pp  16.2% had UUI at 6 months pp  14.6% had mixed incontinence at 6 months pp | Method of birth (SVB, forceps, VE)  Perineal trauma  Episiotomy  Infant birthweight | Univariate associations for exposure measures not reported  Main focus of reporting is on bowel symptoms and associations with 3^rd^ and 4^th^ degree tears |
| Burgio et al,  Obstetrics and Gynecology, 2003 [48] | Prospective birth cohort with follow-up interviews at 6 weeks and 3,6,12 months pp, data on birth events abstracted from hospital medical records | n=523  ‘Convenience sample’ of primparous and multiparous women recruited on postnatal wards, no information regarding response fraction, 94% retention at 6 weeks, 93% at 3 months, 88% at 6 months, 81% at 12 months  Setting: single maternity unit, United States, 1990-1991 | Any urinary incontinence based on 3 questions:  - difficulty controlling urination?  - accidental loss, even small amount?  - ever wet yourself?  5% reported UI prior to 1^st^ pregnancy/birth  59% reported UI in index pregnancy  11.4% had UI at 6 weeks  9.3% had UI at 3 months  10.5% had UI at 6 months  13.3% had UI at 12 months | Parity  Number of vaginal births  Method of birth (index pregnancy): vaginal vs CS; forceps vs no forceps  Episiotomy  Perineal trauma  Length of labour  History of forceps use  BMI  Length of breast feeding  Smoking at baseline  Infant birthweight  Head circumference  Pelvic floor exercises | Univariate associations for all exposures  - UI associated with: vaginal births, forceps in index birth and in prior births, episiotomy, incontinence in pregnancy, smoking, BMI, length of breastfeeding  - no association with infant size, length of labour, perineal trauma or pelvic floor exercises  Multivariate analysis using generalised estimating equations (GEE) to account for repeated measures over 4 time points for total sample  - UI associated with: vaginal births, forceps, smoking, breastfeeding, BMI, incontinence in pregnancy  Second multivariate analysis using GEE restricted to primiparous women:  - UI significantly associated with breastfeeding, symptoms prior to birth, BMI, episiotomy |
| Yip et al,  Neurology and Urodynamics, 2003 [49] | Prospective birth cohort with follow-up telephone interview 4 years after the index birth, data on obstetric events obtained from medical case notes | n=276  Consecutive nulliparous women having vaginal birth, excluding women who had USI in pregnancy or prior to pregnancy, multiple pregnancy or breech delivery; no information on initial response fraction, 53.6% retention at 4 years pp.  Setting: teaching hospital, Hong Kong, 1996 | Urinary stress incontinence: defined as 2 or more episodes in past month, no other information regarding study instrument  25.7% had USI at 4 years pp | Method of birth (SVB, forceps)  Length of labour  Genital tract trauma  Subsequent births  Postpartum urinary retention | Univariate associations for all exposure measures  - none of the obstetric variables associated with USI  Multivariate analysis using logistic regression to assess contribution of subsequent pregnancy to USI adjusting for other obstetric risk factors identified no significant associations |
| Liebling et al,  Am J Obstet Gynecol,  2004 [50] | Prospective birth cohort with follow-up at 6 weeks and 12 months pp no information on source of data on obstetric events (probably case notes) | n=393  Primiparous and multiparous women who had term, singleton, cephalic pregnancies that required operative delivery in surgery at full dilatation  Setting: two maternity units, Bristol, United Kingdom 1999 | Urinary incontinence (not defined) ‘more than occasionally’  16.2% of women who had operative vaginal birth had UI at 6 wks’ & 17% at 12 months pp  2.7% of women who had CS had UI at 6 weeks & 5.4% at 12 months pp | Parity  Method of birth (operative vaginal birth, emergency CS)  Previous difficult delivery  Length of 2^nd^ stage  Epidural  Fetal position  Fetal descent  Operator experience  Infant birthweight  BMI  Maternal age | Univariate associations only reported for method of birth  Multivariate analysis using logistic regression to adjust for potential confounders including all listed exposures:  - increased odds of UI associated with operative vaginal birth( adj OR = 7.8[2.6-23.6] at 6 weeks and adj OR = 3.12 [1.3-7.6] at 12 months) |
| Casey et al,  Am J Obstet Gynecol, 2005 [51] | Prospective birth cohort with follow-up at 5-7 months pp, data on birth events obtained from computerised hospital data base | n=10,643  Nulliparous women recruited in labour, 36% retention at 5-7 months pp (n=3887)  Setting: single maternity unit, Dallas, Texas, 2000-2002 | Urinary stress incontinence: ‘loss of urine when you laugh, cough or sneeze’  Urge incontinence: ‘leak urine when you have urge to urinate, e.g. on way to toilet’  Before pregnancy:  1% USI, 0.7% UUI  at 5-7 months pp:  3.8% USI, 4.2% UUI | Method of birth (SVB, Forceps, CS)  Oxytocin augmentation  Prolonged 2^nd^ stage labour (>2 hours)  Episiotomy  3^rd^ or 4^th^ degree tear  Infant birthweight | Univariate associations for all exposure measures  - CS protective for USI and urge incontinence  - forceps increased risk of urge incontinence  - no other significant associations  Multivariate analysis conducted for outcomes of SUI and urge incontinence:  - significant interaction between CS, oxytocin and epidural for urge incontinence  - no other significant findings, after adjustment |
| Ewings et al,  J Obstet Gynecol, 2005 [52] | Prospective birth cohort recruited on postnatal ward with follow-up at 6 months pp (with nested RCT of pelvic floor exercises), data on obstetric events obtained from questionnaire responses | n=723  Primiparous and multiparous women, excluding women who had stillbirths, very ill babies or prior treatment for urinary incontinence, no information on initial response fraction, 76% participation at 6months pp  Setting: two maternity units, UK, 2001-2002 | Urinary stress incontinence: ‘loss of urine during coughing, laughing, sneezing’  45.5% reported USI at 6 months pp | Parity  Method of birth (Vaginal, CS)  Epidural/spinal  Episiotomy  BMI  Maternal age  Height | Univariate associations for all exposures:  - CS and epidural/spinal protective  - episiotomy increased risk  Multivariate logistic regression adjusting for parity, maternal age, BMI, family history of incontinence, prior symptoms, epidural/spinal (index birth), constipation, all births CS, at least one episiotomy:  - USI significantly associated with: prior symptoms, constipation, episiotomy, recent epidural |
| Glazener et al,  BJOG 2006 [53] | Prospective birth cohort, this analysis based on cross sectional survey at 3 months pp, data on birth events abstracted from case notes or from computerised records | n=3405  Primiparous women with singleton births, 76% response fraction  Settting: maternity units in Aberdeen (Scotland), Birmingham (England) and Dunedin (New Zealand), 1994-1995 | Urinary stress incontinence: ‘lose urine when you cough, laugh, sneeze, run, jump or play sport’  Urge incontinence: ‘urgent desire to pass water and unable to reach toilet in time’  Mixed incontinence: USI and UUI according to above definitions  29% had UI at 3 months pp (of these women 48% had USI, 23% UUI and 30% had mixed UI)  11% reported onset of UI in pregnancy  15% reported onset of UI after birth | Method of birth (SVB, operative vaginal births, CS)  Gestation  Onset of labour  Analgesia in labour  Duration of 2^nd^ stage labour  Total duration of labour  Delivery position  Perineal trauma  Birthweight  Head circumference  Antenatal problems  Maternal age at first birth  Pre-pregnancy BMI  Change in BMI pre-pregnancy to post birth  Prior urinary tract infections  Smoking  Pelvic floor exercises during and after pregnancy | Univariate analyses for all exposure comparisons  - UI associated with method of birth, maternal age, gestation at delivery, infant birthweight, pre-pregnancy BMI  Multivariate analysis using logistic regression restricted to women with onset of UI after birth:  - CS protective cf SVB (adjOR=0.29[0.2-0.4])  - no difference in UI associated with forceps or VE cf SVB  - older women at increased risk of UI  Multivariate analysis using logistic regression restricted to women with persistent antenatal UI (i.e. women who were still incontinent at 3 months pp whose incontinence commenced in pregnancy):  - CS protective cf SVB (adjOR=0.39[0.3-0.6])  - no difference in UI associated with forceps or VE cf SVB  - higher pre-pregnancy BMI (upper two quartiles) increased risk of UI  - higher birthweight infants (upper two quartiles) increased risk of UI |
| MacArthur et al,  BJOG 2006 [54] | Prospective birth cohort with follow-up at 3 months pp and 6 years post index birth, data on birth events abstracted from case notes or from computerised records | n=7879  Primiparous and multiparous women, 72% response fraction to baseline survey at 3 months pp, 54% retention (n=4214) at 6 years post index birth, rate of UI at 3 months pp was similar for respondents and non-respondents (33% vs 34%)  Settting: maternity units in Aberdeen (Scotland), Birmingham (England) and Dunedin (New Zealand), 1994-1995 | Urinary stress incontinence: ‘lose urine when you cough, laugh, sneeze, run, jump or play sport’  Urge incontinence: ‘urgent desire to pass water and unable to reach toilet in time’  Mixed incontinence: USI and UUI according to above definitions  Any incontinence:  33% at 3 months pp  45% at 6 years post index birth  Persistent incontinence:  24% at 6 years post index birth (i.e. women reporting symptoms at 3 months and 6 years) | Parity  Method of birth (SVB, operative vaginal births, CS)  Maternal age at first birth  Ethnic origin (Asian/non-Asian)  For first births:  Onset of labour  Duration of 2^nd^ stage labour  Perineal trauma  Birthweight  Pre-pregnancy BMI | Univariate analyses for all exposure comparisons  Multivariate analysis using logistic regression to assess effects on ‘persistent UI’ of first delivery mode adjusted for maternal age at first birth and number of births:  - CS protective cf SVB (adjOR=0.54[0.4-0.7])  - no difference in UI associated with forceps or VE cf SVB  - older age at first birth and higher parity associated with increased risk of persistent UI    Second multivariate analysis using logistic regression to assess effects on ‘persistent UI’ of first delivery mode adjusted for maternal age at first birth, number of births and additional obstetric variables restricted to primiparous women (at index birth):  - UI less likely among women who had first CS birth (adjOR=0.36[0.3-0.5])  - no associations with forceps/VE or other obstetric variables  - pre-pregnancy BMI in upper quartile (≥25) associated with persistent UI  Third multivariate analysis using logistic regression to assess effects of delivery history in women who had no subsequent births after index birth:  - women who had given birth exclusively by CS less likely to have persistent UI (adjOR=0.46[0.3-0.7])  - no associations for ‘any forceps’ compared with SVB or CS for one but not all births |

**Notes:** CS caesarean section; SVB spontaneous vaginal birth; VE vacuum extraction; UI unspecified urinary incontinence; USI urinary stress incontinence; UUI urinary urge incontinence; pp postpartum; OP occipito-posterior; BMI body mass index
